# From cars to bikes – the feasibility and effect of using e-bikes, longtail bikes and traditional bikes for transportation among parents of children attending kindergarten: design of a randomized cross-over trial

**DOI:** 10.1186/s12889-017-4995-z

**Published:** 2017-12-28

**Authors:** Helga Birgit Bjørnarå, Sveinung Berntsen, Saskia J. te Velde, Liv Fegran, Aslak Fyhri, Benedicte Deforche, Lars Bo Andersen, Elling Bere

**Affiliations:** 1 Department of Public Health, Sport and Nutrition, Faculty of Health and Sport Sciences, University of Agder, Post Box 422, NO-4604 Kristiansand, Norway; 2 Department of Safety and the Environment, Institute of Transport Economics, Gaustadalléen 21, 0349, OSLO, Norway; 3 Unit Health Promotion and Education, Department of Public Health, Faculty of Medicine and Health Sciences, Ghent University, De Pintelaan, 185 Ghent, Belgium; 4 Research unit Physical Activity, Nutrition and Health, Faculty of Physical Education and Physical Therapy, Vrije Universiteit Brussel, Pleinlaan 2, Brussels, Belgium; 5 Faculty of Teacher Education and Sport, Western Norwegian University of Applied Sciences, Sogndal Campus, Bergen, Norway

**Keywords:** Active transportation, E-bike, Cargo bike, Bicycling time, Bicycling distance, Physical activity, Fitness, Body composition

## Abstract

**Background:**

The present study aims to increase bicycling and level of physical activity (PA), and thereby promote health in parents of toddlers, by giving access to different bicycle types. There is a need for greater understanding of e-bikes and their role in the transportation network, and further effects on PA levels and health. Moreover, longtail bikes could meet certain practical needs not fulfilled by e-bikes or traditional bikes, hence increased knowledge regarding their feasibility should be obtained. No previous studies have investigated whether providing an e-bike or a longtail bike over an extended period in a sample of parents of toddlers influence objectively assessed amount of bicycling and total PA level, transportation habits, cardiorespiratory fitness, body composition and blood pressure.

**Methods:**

A randomized cross-over trial will be performed, entailing that participants in the intervention group (*n* = 18) complete the following intervention arms in random order: (i) three months access to an e-bicycle with trailer for child transportation (*n* = 6), (ii) three months access to a longtail bicycle (*n* = 6), and (iii) three months access to a regular bicycle with trailer (*n* = 6), in total nine months. Also, a control group (*n* = 18) maintaining usual transportation and PA habits will be included. A convenience sample consisting of 36 parents of toddlers residing in Kristiansand municipality, Southern Norway, will be recruited. Total amount of bicycling (distance and time), total level of PA, and transportation habits will be measured at baseline and in connection to each intervention arm. Cardiorespiratory fitness, body composition and blood pressure will be measured at baseline and post-intervention. Main outcome will be bicycling distance and time spent cycling.

**Discussion:**

New knowledge relevant for the timely issues of public health and environmental sustainability will be provided among parents of toddlers, representing a target group of greatest importance. There is a call for research on the influence of e-bikes and longtail bikes on travel behavior and PA levels, and whether voluntary cycling could improve health. If the present study reveals promising results, it should be replicated in larger and more representative samples. Eventually, inclusion in national public health policies should be considered.

**Trial registration:**

ID NCT03131518, made public 26.04.2017.

## Background

### Physical activity levels and active transportation

It is well documented that regular physical activity (PA) implies reduced risk for overweight and chronic diseases [1]. Still, one third of adults and four fifths of adolescents globally do not reach PA guidelines [2], and recent European data showed that only 28% of the adult population comply with the PA recommendations [3]. Nonetheless, only ten additional minutes of PA daily would make two thirds of inactive persons adhere to current PA guidelines [4].

For our ancestors, food procurement was inextricably linked to PA and energy expenditure [5], as they needed to hunt and forage in order to get food. Today, this link is broken- we can drive our car to the grocery shop and buy the foods we need with minor energy expenditure. In turn, these changes imply that being physically active requires conscious choices to a larger degree. Physical inactivity is estimated to cause approximately 6–10% of the non-communicable diseases of coronary heart disease, type II diabetes, breast- and colon cancer, and 9% of premature deaths worldwide, i.e. similar health effects as the established risk factors of obesity and smoking [6]. Car use and other forms of motorized transportation favour neither health nor environmental sustainability, as it entails sedentariness and emissions of greenhouse gases (GHGs).

Active transportation, like walking or cycling for transportation purposes, may be a feasible and time efficient way to incorporate PA into daily routines, potentially increasing PA levels [7, 8]. Thus, active transportation could promote health [7, 9–15], prevent obesity [16, 17], and decrease future healthcare costs [18]. It has been calculated that increased PA would translate into significant health gains, entailing major socioeconomic gains for the society [19]. For bicycling specifically, a tripling in cycling in five urban areas in Norway between 2006 and 2009 has been estimated to imply health benefits of 250 million NOK annually [20].

Next to direct effects on fitness and health, an additional advantage of active transportation is the potential to decrease GHG emissions [21, 22], as well as noise and pollution [7]. Currently, motorized transportation is responsible for about 23% of global climate gas discharges [7]. The ambitious goal of the Paris Agreement, entailing carbon neutrality before the end of the century [23], demands initiatives to be generated within all areas of society, not the least within the transportation sector. It is likely that an increased share of travels in Norway could be conducted as active transportation, considering that 25% of daily travels done by car are shorter than 2.5 km [20], and average distance of bicycle trips is 5.1 km [24]. Accordingly, 20% of all travels in the UK are shorter than one mile [25].

### Parents as facilitators of physical activity

Lifestyle behaviors in childhood constitute the foundation for health throughout the lifespan, and research suggests that lifestyle habits, such as PA, track from childhood into adulthood [26, 27]. Likewise, overweight and obese children are more likely to become overweight and obese adults, than normal weight children [28, 29]. Parents are important facilitators and role models of PA for their kids, and among the significant correlates are parental PA, as well as parent participation in child PA [30]. Being transported to kindergarten by bicycle instead of by car could teach children that alternative modes of transport exist, hence representing early adaptation to healthy and sustainable transportation and PA habits. Since parental PA behaviors are crucial for their own and their children’s current and future health, parents of toddlers is a target group of utmost importance. In terms of parental own PA habits, lack of time [31] and stress [32] are repeatedly documented to be negatively associated with PA in adults. In this regard, active transportation could potentially decrease the impact of time scarcity as a barrier, and may also reduce overall perceived stress through incorporating PA into daily transportation, implying less need for additional time-consuming exercise.

### E-bikes and longtails

Electric assisted bicycles (e-bikes) represent an unexploited potential in terms of increased bicycle use. If replacing other motorized modes, and not replacing other PAs, e-bikes could favor both public health and the environment through increased levels of PA and decreased emissions of climate gases [22, 33]. Empirical evidence indicates that mode substitution depends on local context, culture and available transport alternatives, entailing that a larger proportion of car trips is replaced in cardominated countries such as Australia, US and Canada, than in countries with a bicycle culture, such as Denmark and the Netherlands [34]. For illustration, recent Dutch data showed that e-bike ownership reduced car and public transport use, although usage of the traditional bike decreased even more [35]. Nonetheless, e-bike owners reduced their car and public transport use more than traditional bicycle owners [35]. Moreover, in a convenience sample of Norwegian car owners, it was found that those who cycled the least were most interested in buying an e-bike, which in turn could result in mode shifts from cars to bikes [36].

In e-bikes categorized as pedelecs, propulsion is caused partly from the pedal- power of the rider, and partly from an electric engine supplementing power up to 25 km/h, or a maximum power output of 250 watt [34]. It has been claimed that energy efficiency of an e-bike is greater than that of any other mode of transport, except from traditional bicycles [37]. Compared with regular bicycles, e-bikes enable maintenance of speed with less effort, which in turn helps overcoming some of the most common barriers to traditional pedal cycling, such as lack of fitness needed to cycle, hilly terrain, longer distances, lack of time, and lack of end of trip facilities (e.g. change rooms and shower) [34]. Current knowledge suggests that e-bike users cycle more often, and to more distant locations than those using regular bicycles [34, 37, 38], hence possessing greater capacity for exchanging car use than regular bicycles.

A major limitation with traditional bicycles, and also standard e-bikes, is the carrying capacity [39]. At present, there are several different cargo bikes on the market, both human powered and with electric assistance, as well as various bike trailers for carrying goods and/or children. However, carrying stuff on a trailer may be less convenient than directly on a bike. In this regard, so-called longtail bikes possess a great potential, being constructed for carrying an adult, two children and additional goods. Such longtails could possibly promote health through increased cycling, and reduce anthropogenic CO_2_ emissions related to motorized transportation, while simultaneously meeting a practical need not sufficiently accomplished by a traditional bike or e-bike. Considering that a great share of car travels are done within a limited range [20, 25], longtail bikes might represent a feasible mode of transportation, yet current scientific evidence is scarce. Still yet, our research group has tested human powered longtail bikes in different families for periods lasting up to five years, elucidating the potential of longtails for various trip purposes, and for all seasons and weather conditions. In line with this, American data support possible mode substitution and a decline in car travel among cargo bike owners [40]. Giving specific attention to females with children, representing a minority group in the bicycling community, Schwartz and Riggs [41] newly suggested that under certain circumstances and subject to a certain culture, the cargo bike has emerged as an option for women to choose.

### Health effects of e-bicycling and use of longtails/transport bikes

It is repeatedly found that both active [42] and inactive [43, 44] subjects reach PA levels sufficiently high to meet the moderate-to-vigorous-intensity standard (3–9 metabolic equivalents (METs)) of the physical activity guidelines [1, 45], when e-biking. Although e-bikes seem to entail lower intensity than traditional bikes [46], e-bikes could still boost overall levels of PA [34] and thereby promote health, if combined with more frequent and longer trips as proposed [37, 38]. Current evidence regarding health effects of commuting with an e-bike is scarce. De Geus and colleagues [47] conducted a quasi-experimental study in twenty untrained men and women, who were provided with an e-bike for six weeks. No change in maximum oxygen uptake (VO_2_ max) was found, yet a significant gain in maximal power output was achieved after six weeks of e-biking [47]. A recent Norwegian pilot study equipped 25 inactive adults with an e-bike for eight months, measuring participants′ VO_2_ max at baseline and at intervention determination [48]. Results showed an average 8% improvement in VO_2_ max, and cycling distance was positively associated with the increase, yet no control group was included. Focus group interviews were conducted and analyzed as well, revealing that e-biking contributed to highly positive experiences regarding active commuting (unpublished results). Moreover, a recent American study in twenty sedentary commuters reported four weeks of pedelec commuting to result in significant improvements in 2-h post-plasma glucose levels, VO_2_ max and maximal power output [49]. In addition, no compensatory changes in overall PA levels were observed, hence pedelec commuting helped the participants to meet PA recommendations [49]. In terms of potential health effects of using human powered longtail bikes or other cargo bikes, no previous studies have addressed these associations. It seems reasonable to assume though [46], that the intensity will be higher than for traditional bikes due to the weight of the cargo, entailing additional health effects if used as frequently as traditional bikes.

### Accessibility, social support and intrinsic motivation

When developing programs aiming to promote certain behaviors, such as bicycling with e-bikes and longtail bikes, relevant determinants for the behaviors of interest should be known. Existing literature and behavioral theories provide relevant determinants at several levels, that is, the personal (e.g. motivation, intention, knowledge, attitude), interpersonal (social support, modelling) and/or environmental (availability, accessibility, policy) level. The socio-ecological framework suggested by Sallis et al. [50] describes most of these determinants, especially the environmental determinants, while theories such as the Social cognitive theory [51] and the Self-determination theory [52] describe the more interpersonal and cognitive processes to increase goals and motivation for various behaviors, like PA. The Self-determination theory focuses on diverse types of motivation, and argues that intrinsic motivation should be strived for, in order to result in sustained behavior [52]. According to the socio-ecological framework [50], accessibility is one important environmental determinant for PA, including active transportation. Supporting this, a recent British study indicated that when made accessible, e-bikes could facilitate active travel and have substantial effects on travel behavior, also in subjects traditionally undertaking less PA or feeling unable to use a conventional bike [53]. In total 80 employees were loaned an e-bike from two major employers in Brighton, during a six- to eight-week trial period. Across all participants 75% chose to use the e-bike at least once a week, car mileage was reduced by 20%, and 59% of the employees reported that their overall PA increased. At the end of the trial, 73% of the employees said they would cycle to work at least once a week if they had an e-bike available [53]. Bike share programs may be considered another aspect of accessibility, and a number of cities have now introduced e-bike shares, potentially encouraging new users to bike share [54]. Underpinning this, a pilot study trialing a university based e-bike share in North-America reported that new users were attracted to cycling [55]. Multi-city analyses of regular bike shares’ impact on car use and PA suggest that car use decreases, yet of limited magnitude [56]. Nonetheless, PA levels could increase [57] due to mode shifts, likely resulting in overall positive health effects [58]. Another important behavioral interpersonal determinant described in the socio-ecological framework is social support, and the workplace environment, entailing both the social and the physical environment, could facilitate active transportation [50]. A previous study by Wen and colleagues [59] assessing the role of workplaces in promoting active commuting, reported a significant inverse association between employees’ perception of workplace encouragement for active travel and driving to work. Also, physical support at work, such as available bike parking and presence of showers, as well as cultural and social support for active transportation, has shown to be relevant for female employees’ transport choices [60]. Accordingly, Yang et al. [61] found that worksite support and policies tended to associate with active commuting and the use of public transit. Grounded in this, together with previously reported higher income and education among e-bike owners [62], it is reasonable to assume that initiatives providing bike accessibility could increase cycling. In line with Self-determination theory, we hypothesize that increased accessibility and social support could facilitate intrinsic motivation for biking [52] through meeting the basic psychological needs, i.e. feelings of autonomy, competence and relatedness [63], which in turn could result in higher levels of bicycling.

### Objectives

We aim to assess the effect of an intervention where participants will be provided access to an e-bike (including a trailer), a longtail bike and a traditional bike (including a trailer), each bike for three months, on the following aspects:

#### Primary objectives

1. Objectively assessed amount of bicycling (distance and time) and total time of moderate-to-vigorous intensity PA (MVPA), assessed at baseline, after three months, six months and nine months (post-intervention).
2. Mode shifts from car to bike, assessed at baseline, after three months, six months and nine months (post-intervention).

#### Secondary objectives

1. Cardiorespiratory fitness (VO_2_ max), blood pressure and body composition, measured at baseline and after nine months (post-intervention).
2. Self-reported health and health-related quality of life (HRQoL), assessed at baseline, after three months, six months and nine months (post-intervention).
3. Experiences with bicycling and intrinsic motivation for bicycling, explored after three months, six months and nine months (post-intervention).

#### Other study objectives

4. How season and weather conditions influence amount of bicycling.
5. Potential spill-over effects on participants’ partners.

## Methods

### Study design

The present study will have a cross-over design, entailing that participants in the intervention group (*n* = 18) will complete the following intervention arms in random order: (i) three months access to an e-bike with trailer (*n* = 6), (ii) three months access to a longtail bike (*n* = 6), and (iii) three months access to a traditional bike with trailer (*n* = 6), in total nine months. Also, a control group (*n* = 18) maintaining usual transportation and PA habits will be included. Randomization of participants into intervention or control group will be stratified according to sex and cardiorespiratory fitness. As incentive for participants randomized into the control group, those who fulfill the study will be in the draw of one traditional bicycle, including a trailer. To reduce the risk of accidents and injuries, bike helmets (parent and child), a reflex vest, lights and winter tyres with spikes will be handed out. The present study was approved by The Norwegian Social Science Data Services (NSD), and all participants will provide informed consent prior to study start. The trial is registered at clinicaltrials.gov, with number NCT03131518.

### Study sample

A convenience sample consisting of 36 parents of toddlers residing in Kristiansand municipality, Southern Norway, will be recruited. Main outcome will be total amount of bicycling (distance and time). Due to few previous studies in e-bikes and none in longtail bikes targeting total amount of bicycling, power calculations are challenging to perform. However, based on a standard deviation (SD) of 60 min/week [48], a power of 0.80 and a significance level of 5%, we will be able to detect an increase in cycling time from 15 min (based on inclusion criteria) to 75 min a week (i.e. half of weekly PA recommendations) with 16 subjects in the intervention group and 16 subjects in the control group. Yet, to account for 10% drop-out, and to utilize the bikes optimally according to the study design (see Table 1), we will include 18 subjects in both groups (intervention and control). Inclusion criteria are: (i) being able to understand and read Norwegian, (ii) having one child born in year 2013, 2014 or 2015, attending kindergarten, (iii) being responsible for bringing/picking up the child in the kindergarten ≥5 times per week/at least half of the times, (iv) residing 2–10 km from the workplace, (v) residing <3 km from the kindergarten and the grocery shop, (vi) having car-access (vii) possessing a smartphone requiring personal pin code or comparable safety solution for usage, (viii) being between 167 and 190 cm tall (due to the size of accessible bikes), and (ix) have the opportunity to store the bikes indoors. Exclusion criteria are: (i) being physically active, i.e. meeting the PA recommendations [1, 45], (ii) having bicycled more than once weekly during the last twelve months to the workplace, kindergarten or the grocery shop, and (iii) suffering from severe cardiovascular diseases or upper respiratory tract diseases.

**Table 1:** Possible combination of intervention arms

Month 1–3	Month 4–6	Month 7–9	
E-bike	Longtail	Traditional	*n* = 3
E-bike	Traditional	Longtail	*n* = 3
Longtail	E-bike	Traditional	*n* = 3
Longtail	Traditional	E-bike	*n* = 3
Traditional bike	E-bike	Longtail	*n* = 3
Traditional bike	Longtail	E-bike	*n* = 3

### Measurements

#### Questionnaire survey

When signing up and providing consent electronically, parents will supply relevant background information such as gender, age, ethnicity, education, income and occupational status, and information allowing to determine eligibility for inclusion. Moreover, a web-based questionnaire will assess transportation habits, self-perceived health and HRQoL [64], and determinants for bicycling, e.g. intrinsic motivation [52], at baseline and post all intervention arms, i.e. in total four times. Participant’s partners will be requested to fill in the questionnaire at baseline and post-intervention, to assess potential spill-over effects.

#### Bicycle use and total physical activity level

Total amount of bicycling (i.e. cycling distance, time and pace) will be measured with a cycle computer and an appropriate smartphone application, the latter being developed at UiA in cooperation with Department of Information and Communication Technology. The app shall include a diary-function, enabling registration of bicycle type (el/longtail/traditional) and trip purpose. Total time of MVPA will be measured for seven consecutive days at study start and connected to each intervention arm (at 3, 6 and 9 months), using the monitor SenseWear Armband Mini (SWA; BodyMedia, Pittsburgh, Pennsylvania, USA). Data will be downloaded using the software SenseWear Professional V.8.1 (BodyMedia, Pittsburgh, Pennsylvania, USA), with a valid day defined as at least 80% (19.2 h) wearing time. To be included in the analyses, participants need to provide a minimum of four valid days, comprising at least one weekend day [65, 66]. The cut-off defining MVPA will be 3 METs [67]. In addition, seasonal variations in bicycling amount will be explored, and weather data (temperature, rainfall, snow, etc.) will be collected.

#### Physiological parameters

At baseline and post-intervention, cardiorespiratory fitness (VO_2_ max, minute ventilation (VE) and respiratory exchange ratio (RER)) will be measured performing treadmill walking/running to exhaustion, using a modified Balke-protocol according to Edvardsen et al. [68]. Heart rate (HR) will be registered every minute using the heart rate sensor Polar M400 (Polar Electro, Oy, Kempele, Finland). Time to exhaustion will be measured as minutes from start to test completion, i.e. reaching VO_2_ max. Criteria for acceptable VO_2_ max will be determined according to sex and age, as described by Edvardsen et al. [69]. In order to measure body composition (i.e. the distribution of fat mass, non-bone lean mass and bone mineral density) rapid and accurately, dual-energy X-ray absorptiometry (DXA; GE-Lunar Prodigy, Madison, WI, USA) will be used [70, 71]. In addition, blood pressure will be measured with the monitor Microlife BP A100 (Widnau, Switzerland), and body weight and height (height only at baseline) will be measured to the nearest 0.1 kg and 0.5 cm, respectively, with participants wearing light clothes and no shoes. Oral and written instructions will be provided prior testing, and all measurements will be performed by the same test leaders and in the same order each time.

#### Qualitative interviews

Apart from the influence of accessibility on total amount of bicycling [50], and the feasibility of bicycling for transportation, we also want to address intrinsic motivation as one relevant determinant, guided by Self-determination theory (SDT) [52]. Thus, participants in the intervention group (*n* = 18) will be invited to take part in semi-structured qualitative interviews after three months, six months and at study completion. Data collection method will be focus group interviews (*n* = 6 in each group, combining individuals using the same type of bike), to obtain a nuanced investigation of the aspects of interest, based on group discussions and interactions between the participants [72]. To examine the feasibility of using different bicycle types for transportation, main focus for the interviews will be exploration of participants’ experiences with usage of the different bicycle types, and further intrinsic motivation [63] for bicycling. One trained interviewer (with public health background) will lead the interviews, while an assistant will be present for observation.

### Quantitative and qualitative data analyses

The statistical analyses for the quantitative data will be performed using the statistical software package IBM SPSS Statistics version 24.0 (IBM Corp., Somers, New York, USA). A two-sided *p*-value of <0.05 will be considered statistically significant. Descriptive analyses will be conducted and continuous variables will be presented as means and standard deviations (SD), categorical variables as proportions. Moreover, regression analyses will be performed according to Twisk and Proper [73], using data from the post-measures adjusting for baseline measures, to assess differences between (1) the control group and the intervention group (between design) for all outcome variables, and (2) within the intervention group when comparing the three different arms regarding total amount of bicycling and PA, transportation habits, and self-reported health and HRQoL.

A qualitative content analysis will be conducted from a phenomenologic-hermeneutic perspective [74]. Recordings of the qualitative interviews will be transcribed verbatim and read repeatedly to grasp the meaning of the data as a whole. Data will then be imported into the software analysis program NVivo 11 for further analysis. The next step will be to separate the text into meaning units; i.e. the words, sentences or paragraphs containing aspects related to each other through their content and context. Following this, the meaning units will be classified into subcategories, by pooling data with similar characteristics together in a category defined by its content. Finally, the subcategories will be combined and categorized into main categories.

## Discussion

Active transportation with traditional bicycles has shown potential to increase PA levels [7, 8] and reduce climate gas emissions related to car use [21], thereby promoting public health and environmental sustainability. E-bikes are becoming increasingly popular, yet current knowledge is sparse regarding influence of e-biking on travel behavior and total levels of PA [34]. The feasibility of e-bikes as mode of transport among parents of toddlers should be explored, considering the impact of parental PA and transportation habits on their children’s future habits. Also, there is a call for increased understanding whether voluntary cycling with e-bikes could improve health. In addition to e-bikes, longtail bikes could possibly contribute positively to both increased bicycling in everyday life, as well as a shift from cars to bikes, as they may meet needs related to transportation of children and goods not sufficiently accomplished by traditional bikes or e-bikes. However, this likely potential of longtail bikes has not previously been scientifically explored. Therefore, the project “From cars to bikes” aims to assess the feasibility and effect of an intervention providing participants free access to an e-bike (including a trailer), a longtail bike, and a traditional bike (including a trailer) for an extended period of in total nine months. Study objectives of the present project are in accordance with aims presented in international and national strategic documents, such as the Paris Agreement [23], the Norwegian National Transportation plan 2018–2029 [75], the latest Norwegian White Paper on public health, “Mastering and opportunities” [76], and both national [77] and global [1] recommendations for PA. Hence, “From cars to bikes” will add new knowledge to topical issues related to public health and environmental sustainability in a target group of utmost relevance, i.e. parents of toddlers.

The strengths of the present study are firstly that the intervention is being conducted in a natural setting with accessibility to different bicycle types representing the intervention arms, and no instructions in terms of bicycling amount. Thus, the effect of accessibility on voluntary cycling (distance, time and pace) and potential health effects can be examined - in other words, both effectiveness and efficacy [78]. As discussed herein, we need increased knowledge regarding potential health effects (efficacy) of using e-bikes and longtail bikes for transportation. Still yet, public health and environmental gains will be attenuated if it turns out that bicycling for transportation does not represent a feasible mode of transport for parents with children attending kindergarten, and “compliance” (bicycling) is low (effectiveness), meaning that both effectiveness and efficacy are relevant aspects. In this regard, inclusion of semi-structured qualitative interviews will enrich the study, as it allows for an exploration of participants’ experiences and pros and cons with bicycling for transportation, when using different bicycle types. An additional advantage is that the cross-over design (within design) removes variability between subjects and thus increases power, compared with a parallel group design (between design). Also, as opposed to a between design, comparability of subjects within conditions is enabled. Further, the use of a reference method for assessing body composition (DXA), and objective measures of total levels of MVPA (SWA) and amount of bicycling (cycle computer and smartphone-app), are study strengths.

Nonetheless, there are some study limitations. The intervention is quite straightforward, addressing accessibility as one main behavioral determinant, yet reality is more complex. If other determinants are equally or even more important than accessibility, we may not achieve behavior change due to exclusion of these other determinants. Moreover, if effectiveness comes out low, i.e. parents are not bicycling, we will not obtain enough data to draw any conclusions regarding potential health effects from bicycling for transportation. Also, sample size and study power are shortcomings; we expect to detect significant differences in the physiological parameters between the control and intervention group, yet not between the three intervention arms. Still, relevant knowledge regarding total amount of bicycling (main outcome) and PA, as well as travel habits and motivation, is likely to be acquired across the bicycle types. It should be mentioned though, that period effects and carry-over effects could influence these latter aspects, due to the lack of wash-out periods between the intervention arms. Besides, the cross-over design requires a longer study period than a parallel group design, which may increase the drop-out risk. And, because we are recruiting a convenience sample, self-selection and homogeneity is likely to impair generalizability.

## Conclusion

There is a call for research on the influence of e-bikes and longtail bikes on travel behavior and PA levels, together with increased understanding whether voluntary cycling with different bicycle types could improve health. Attempting to meet this knowledge gap, the present study will address these topics and provide new knowledge relevant for the timely issues of public health and environmental sustainability in a target group of greatest importance, that is, parents of toddlers. If the present study reveals promising results, it should be replicated in larger and more representative samples, enabling a comparison of health effects across the different bicycle types as well. Eventually, inclusion in national public health policies should be considered.

## Abbreviations

DXA: dual-energy X-ray absorptiometry
E-bikes: electric assisted bicycles
GHGs: greenhouse gases
HRQoL: health-related quality of life
METs: metabolic equivalents
MVPA: moderate-to-vigorous physical activity
PA: physical activity
SWA: SenseWear Armband
VO_2_ max: maximum oxygen uptake
